# Longitudinal Estimation of Adequate Physical Activity Levels to Reduce the Risk of Dementia in Older Adults with MCI

**DOI:** 10.1093/geroni/igaf122.2856

**Published:** 2025-12-31

**Authors:** Jungjoo Lee, Junhyoung Kim, Donghyuck Cho, Min Jun Hwang

**Affiliations:** Texas A&M University, Texas A&M University, Texas, United States; Texas A&M University, College Station, Texas, United States; Cranbrook Academy of Art, Bloomfield Hills, Michigan, United States; Phillips Exeter Academy, Exeter, New Hampshire, United States

## Abstract

This study estimated the level of physical activity (PA) required to reduce the risk of Alzheimer’s disease and related dementias (AD/ADRD), focusing on identifying the adequate PA level to achieve significant risk reduction among older adults with Mild Cognitive Impairment (MCI). This longitudinal study investigated Health and Retirement Study data between 2012 and 2020. The sample included 9,714 individuals with MCI, screened using the Telephone Interview for Cognitive Status-27 at baseline in 2012. The dependent variable was the diagnosis of AD or ADRD between 2012 and 2020. To ensure reliability, this study included participants who responded to the diagnosis questions in a consistent chronological order. The independent variable was the average level of PA participation, including activities such as exercise and walking, from 2012 to 2020. Covariates included age, sex, education, and cognitive function. A Generalized Estimating Equation was used for analysis. Results indicated that the risk of AD/ADRD decreased by 3% at a PA level of 2.20 (OR = 0.970, 95% CI [0.958, 0.983]) and exhibited a linear declining trend with increasing PA levels: a 3.3% decrease at 2.40 (OR = 0.967, 95% CI [0.956, 0.979]), a 3.8% decrease at 2.60 (OR = 0.962, 95% CI [0.948, 0.975]), and a 4.6% decrease at 2.80 (OR = 0.954, 95% CI [0.946, 0.964]). Engaging in exercise and walking at least twice a week was identified as the threshold for achieving a significant reduction in the risk of AD/ADRD, with greater PA engagement further enhancing risk reduction in older adults with MCI.

